# Hub Gene and Its Key Effects on Diffuse Large B-Cell Lymphoma by Weighted Gene Coexpression Network Analysis

**DOI:** 10.1155/2021/8127145

**Published:** 2021-11-27

**Authors:** Chao Ma, Haoyu Li

**Affiliations:** ^1^Department of Ophthalmology, The First Affiliated Hospital of Zhengzhou University, China; ^2^The Eye Hospital, School of Ophthalmology and Optometry, Wenzhou Medical University, China; ^3^Glaucoma Research Institute, Wenzhou Medical University, China; ^4^National Clinical Research Center for Ocular Diseases, China

## Abstract

Diffuse large B-cell lymphoma (DLBC) is a kind of tumor with rapid progress and poor prognosis. Therefore, it is necessary to explore new biomarkers or therapeutic targets to assist in diagnosis or treatment. This study is aimed at screening hub genes by weighted gene coexpression network analysis (WGCNA) and exploring the significance of overall survival (OS) in DLBC patients. Statistical data using WGCNA to analyze mRNA expression in DLBC patients came from The Cancer Genome Atlas (TCGA) dataset. After analyzing with clinical information, the biological functions of hub genes were detected. Survival analysis, Cox regression detection, and correlation analysis of the hub genes were carried out. The potential function of the hub gene related to prognosis was predicted by gene set enrichment analysis (GSEA). The results showed that *APOE*, *CTSD*, *LGALS2*, and *TMEM176B* expression in normal tissues was significantly higher than that in cancer tissues (*P* < 0.01). Survival analysis showed that patients with high *APOE* and *CTSD* were associated with better OS (*P* < 0.01). *APOE* and *CTSD* genes were mainly enriched in the regulation of ROS and oxidative stress. The two hub genes related to the prognosis of DLBC were identified and verified based on WGCNA. Survival analysis showed that the overexpression of *APOE* and *CTSD* in DLBC might be beneficial to the prognosis. These findings identified vital pathways and genes that may become new therapeutic targets and contribute to prognostic indicators.

## 1. Introduction

Diffuse large B-cell lymphoma (DLBC) is one of the most common types of invasive non-Hodgkin's lymphoma (NHL), accounting for about 25% of NHL cases [1]. DLBC is also a heterogeneous malignant tumor in biology and clinic; the pathology and mechanism of DLBC remain unclear, with about 40 percent of patients dying from the lymphoma. Therefore, the study of genes and signal pathways regulated during tumorigenesis will contribute to the study of the pathological mechanism of DLBC and guide the therapeutic effect [2, 3]. It usually occurs only outside the central nervous system or is less isolated from the central nervous system [4]. It is very aggressive and progresses rapidly, so early treatment is the critical way to save the lives of DLBC patients. Recently, studies have shown that the shorter the interval between diagnosis and treatment, the more helpful it is to improve the survival rate of DLBC patients [5, 6]. In addition, some crucial genes and pathways have been identified; these genes and pathways have made significant progress in the diagnosis and treatment of DLBC [7, 8]. At present, there are no specific biomarkers widely used in the clinic for DLBC, which limits the understanding of the pathogenesis of the disease and the predictive risk factors of disease prognosis.

Rich chips and sequencing information from genome technology, such as The Cancer Genome Atlas (TCGA), provide meaningful opportunities to discover new diagnostic or therapeutic targets [9]. TCGA database was established in 2006. The purpose of the database is to collect and analyze clinical and laboratory molecular data of different tumor types by sampling cases of different tumor types. At present, the atlas is the most comprehensive storage database of human cancer molecular and clinical data [9]. Mining, integrating, and reanalyzing the data stored in public databases can provide a theoretical basis and valuable clues for new research. Moreover, we can identify tissue-specific biomarkers and their key-related pathways based on bioinformatics methods and expression profiles.

Weighted gene coexpression network analysis (WGCNA) is often used to explore the complex relationship between genes and phenotypes. The significant advantage is that WGCNA converts gene expression data into a coexpression module, which provides a basis for in-depth mining of phenotypic features of interest [10, 11]. WGCNA focuses on gene modules rather than single genes [12]. It simplifies the interpretation of thousands of gene responses that synthesize genomes or modules. Network analysis establishes the relationship between genes. If the expression of genes is related, it proves that they are interrelated. Through different weights, genes can be more or less closely linked. The connections between genes are then interpreted as different distances, which are used to group genes into modules. In conclusion, it depends on the assumption that all highly related genes in a module participate in a common biological process. It is widely used in various biological processes, such as cancer, genetics, and brain imaging data analysis, and is very helpful in identifying candidate biomarkers or therapeutic targets [13, 14]. Joint follow-up bioinformatics analysis methods can help compare the process of differentially expressed genes and help to understand the interaction between genes in different coexpression modules.

Gene enrichment analysis and predictive analysis are helpful to find targets for disease intervention and understand the mechanism of disease development. This present study is aimed at identifying the potential treatment target and prognostic markers for DLBC via constructing a coexpression module using DLBC expression data in TCGA. The hub genes in each module were identified, and the function and pathway correlation analysis was carried out to help determine the function of these significant module genes.

## 2. Materials and Methods

### 2.1. Expression Analysis of Microarray Data of DLBC Samples

The mRNA sequencing data and clinical characteristics of 48 patients with DLBC were downloaded from TCGA database (https://cancergenome.nih.gov/, accessed by August 2, 2019). Fragments Per Kilobase of transcript per Million fragments mapped (FPKM) method was used to encapsulate the data, and then, the mRNA sequencing data annotation information was used to match the probe with the corresponding gene to transform the gene name into gene symbol. The threshold was determined by the number of genes with different expression thresholds. The first 25 percent of the most mutated genes (3,218 genes) were included for further analyses. WGCNA algorithms were used to evaluate gene expression values [15].

### 2.2. Analysis of the Construction of DLBC Coexpression Module

The power value was screened out in the process of module construction by using the WGCNA algorithm. The gradient method was used to test different modules' independence and average connectivity with different power values (ranging from 1 to 30). When the degree of independence was 0.8, the appropriate power value was determined. Then, the soft threshold test was performed. Once the power value was determined, the module construction clustering was carried out according to the WGCNA algorithm, and then, the corresponding gene information was taken out.

### 2.3. Construction of Coexpression Module of DLBC and Clinical Data

A thermal mapping kit was constructed by R v3.6 to analyze the intensity of the interaction. Then, the correlation between the characteristics of the module-trait association module and clinical traits was visually expressed. The upper value in each color module indicates the correlation, and the value in the parentheses below indicates the *P* value. For each expression profile, the gene significance (GS) was calculated as the absolute value of the correlation between the expression profile and traits, and the module membership (MM) was defined as the correlation between the expression profile and each module Eigengene. The relationship between expression profile and traits was analyzed to make a scatter plot between GS and MM [14].

### 2.4. Hub Genes Identification and Functional Analysis

The module data in WGCNA was imported into Cytoscape software, and the “cytoHub” plug-in was used to screen the hub genes. Boxplots of mRNA expressions were generated depending on the Gene Expression Profiling Interactive Analysis (GEPIA: http://gepia.cancer-pku.cn/, accessed by August 1, 2019) dataset [16]. The selected hub genes were analyzed by Gene Ontology (GO) and Kyoto Encyclopedia of Genes and Genomes (KEGG) analysis using Database for Annotation, Visualization, and Integrated Discovery (DAVID v.6.8: https://david.ncifcrf.gov/, accessed by August 2, 2019) [17, 18]. The possible functions of the hub gene were analyzed by biological process (BP), cellular component (CC), and molecular functional (MF), and the possible signal pathways were analyzed by KEGG. Then, the Biological Networks Gene Ontology (BiNGO) plug-in of Cytoscape was used to predict the function of the genes.

### 2.5. Construction of Hub Genes Protein-Protein Interaction (PPI) and Genetic Interaction (GI) Network

The PPI network was used to analyze the hub genes at the protein level, and the Search Tool for the Retrieval of Interacting Genes/Proteins (STRING v11.0: https://string-db.org/, accessed by August 1, 2019) was used to check and predict the interaction between proteins [19]. The GI network using gene function prediction was constructed to understand the complex interactions between genes. We used Gene Multiple Association Network Integration Algorithm (GeneMANIA https://genemania.org/, accessed Aug. 1, 2019) to analyze the hub genes. The statistical significance was expressed as a collective score of >0.15.

### 2.6. Survival Analysis and Cox Regression

According to the 50th percentile cut-off value of each hub gene mRNA, the patients were divided into the high-expression and low-expression groups. Log-rank test and Kaplan-Meier estimation were performed to obtain log-rank *P* value and evaluate hub genes in OS. Cox regression analysis was performed to determine the relationship between risk score and clinical information and generate a nomogram. The survival curve and nomogram were carried out by R v3.6.

### 2.7. mRNA Correlation Analysis

The Pearson correlation coefficient was generated by R v3.6 to evaluate the coexpression relationship between hub genes.

### 2.8. Gene Set Enrichment Analysis (GSEA)

GSEA (http://software.broadinstitute.org/gsea/index.jsp; accessed by August 1, 2019) [20] is a computational method used to assess whether a priori defined group of genes shows statistical significance or a consistent difference between the two biological states. We used two hub genes (*APOE* and *CTSD*), which impacted prognosis, to divide DLBC patients' data into the high-risk and low-risk groups. GSEA was used to quantify the genes' up- and downregulation according to folding changes. If most of the gene sets showed high-expression and high-risk scores, the gene sets would show a positive enrichment score called enrichment. The gene sets used in this study (c2.cp.kegg.v5.2.symbols.gmt) could be downloaded from the Molecular Signature Database (MSigDB; http:/software.wide http://stitute.org/gsea/msigdb/index.jsp), and FDR < 25%, NES > 1, and nominal *P* < 0.05 were regarded as statistically significant.

### 2.9. Statistical Analyses

R v3.6 was used to generate the correlation graph, survival curve, nomogram, and data visualization. Additionally, *P* < 0.05 was statistically significant unless otherwise indicated.

## 3. Results

### 3.1. Construction and Screening of DLBC Coexpression Module

**Figure 1:**
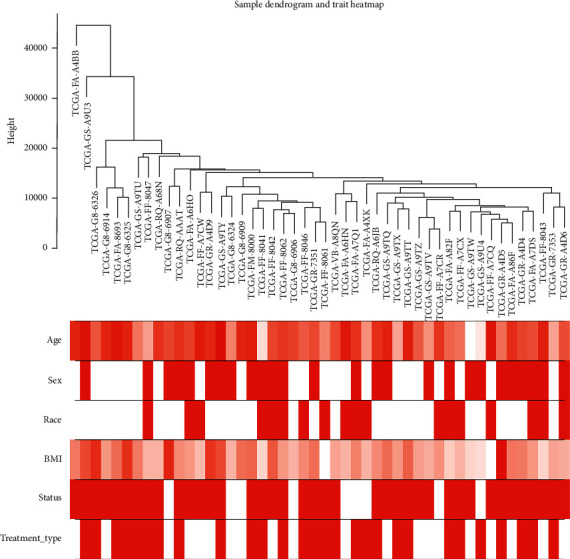
Clustering tree in WGCNA, clustering tree of 48 samples of DLBC extracted from TCGA database.

**Figure 2:**
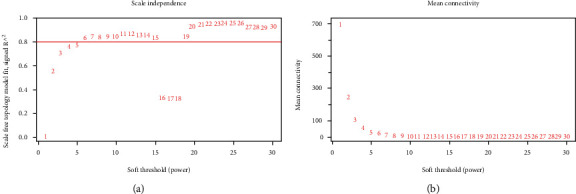
Network topology analysis with different soft threshold power. (a) The scale-free fitting index as a function of the soft threshold power. (b) The average connectivity as a function of the soft threshold power.

**Figure 3:**
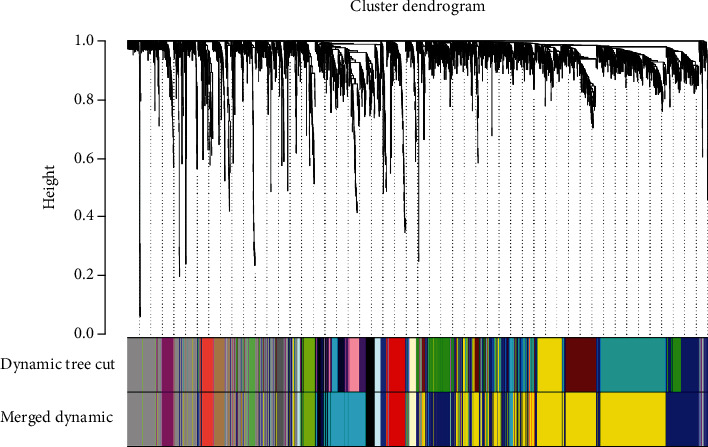
In the clustering tree of genes, different colors are assigned to different modules through topological overlap calculation. After merging similar modules, a total of 15 coexpression modules were constructed, which were displayed in different colors.

**Figure 4:**
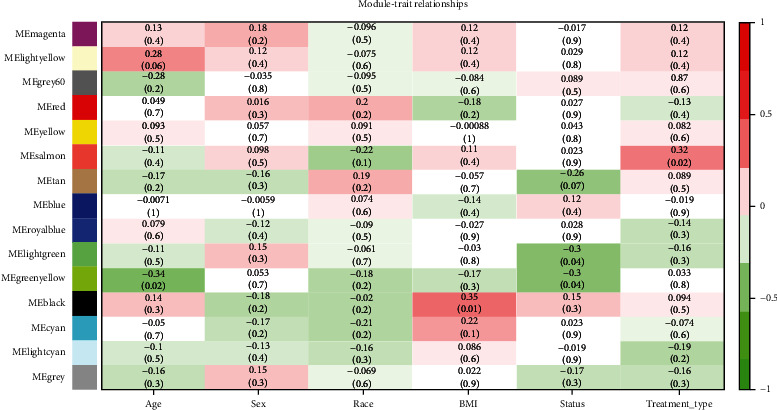
Module-trait association. Correlation thermography between modular feature genes and clinical features of DLBC. Each row corresponds to a module feature, and the column corresponds to a feature. Each cell contains the correlation and the corresponding *P* value.

**Figure 5:**
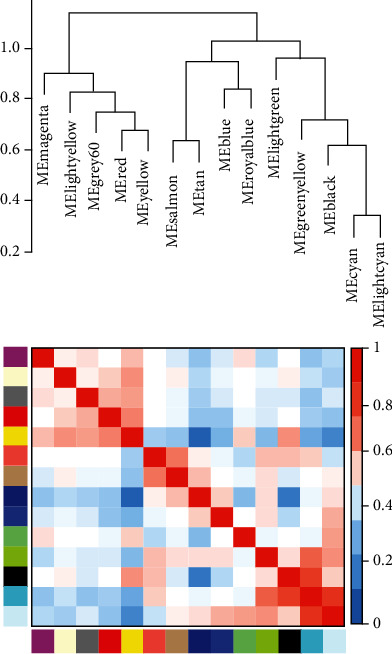
Eigengene tree view and Eigengene adjacent heat map. At the top are the color names of the 14 gene modules, and at the bottom, the correlation varies depending on the color.

**Figure 6:**
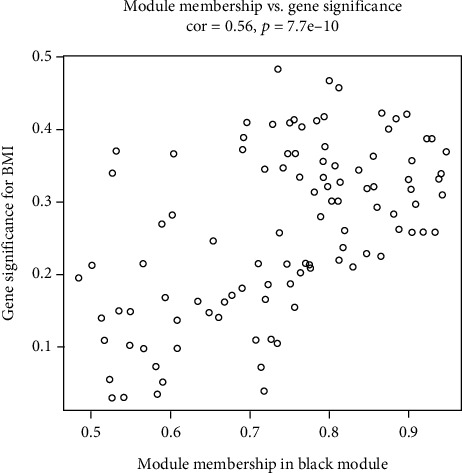
The scatter plot of the correlation for BMI-related gene between module membership and gene significance in the black module.

In this study, we obtained the DLBC dataset in TCGA, 48 sample expression matrices. Then, we selected the first 25% of the genes (3,218 genes) for WGCNA to identify modules highly related to clinical information. First, we observed and clustered the samples to detect the number of gene expressions in different traits. Red indicated more gene expression, less white, and gray indicated deletion. No deletions were found in this study (Figure 1). Then, the soft threshold (power value) was calculated, and when the weight was equaled to 10, the independence exceeded 0.8 and had higher average connectivity (Figure 2). Using this power value for hierarchical clustering analysis and combining similar analysis results, 14 different gene coexpression modules were identified in DLBC (Figure 3). After docking with clinical character data, it was found that there was a significant correlation between the black module and body mass index (Figure 4). The Eigengene tree was used to show the correlation between the gene and the module between the phase Eigengene groups (Figure 5). Finally, we conducted a scatter diagram of the correlation between the black module and genetic characteristics (Figure 6).

### 3.2. Hub Genes Identification and Functional Analysis

**Figure 7:**
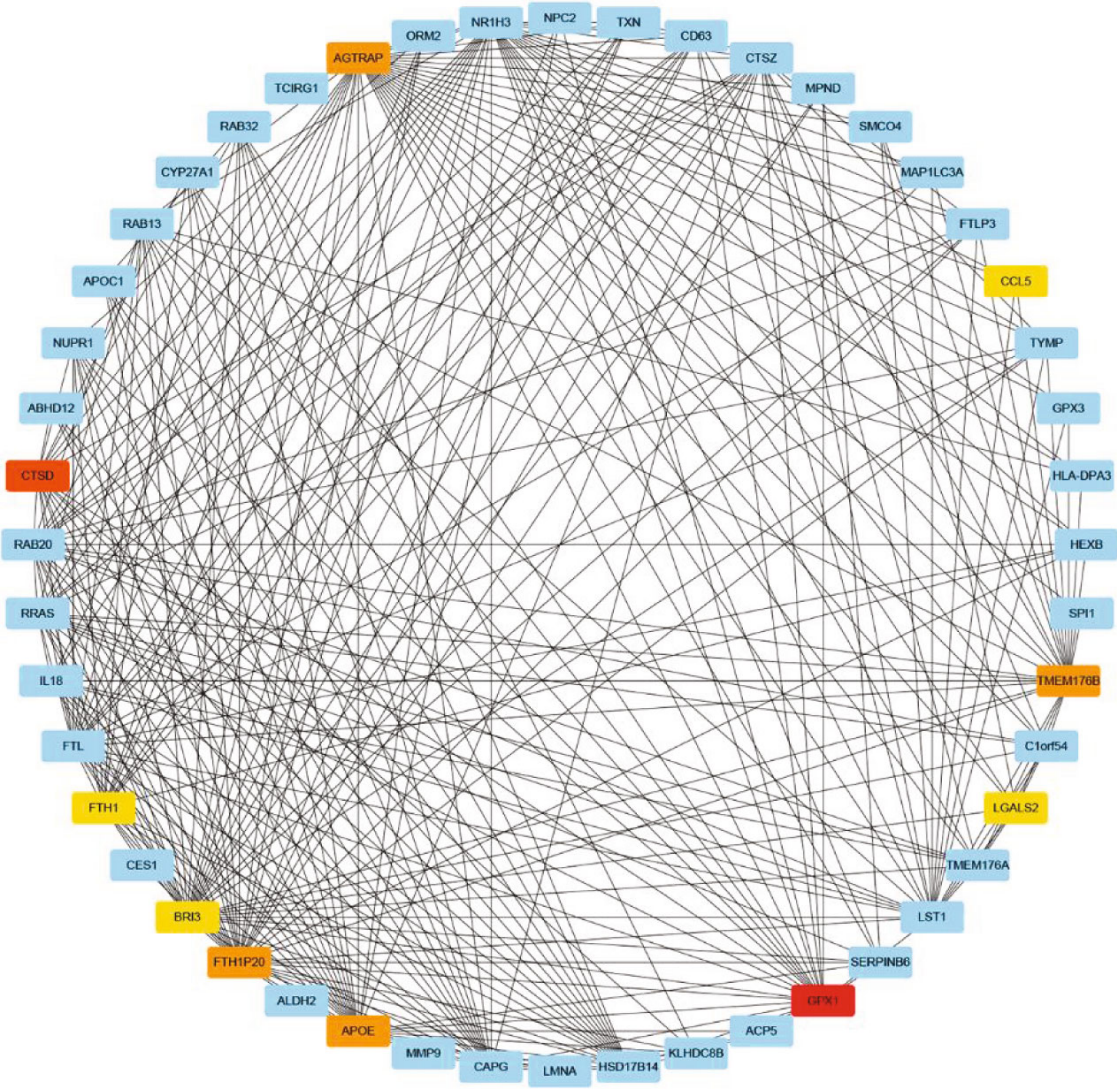
The cytoHub software package shows the top 10 hub genes in the black module.

**Figure 8:**
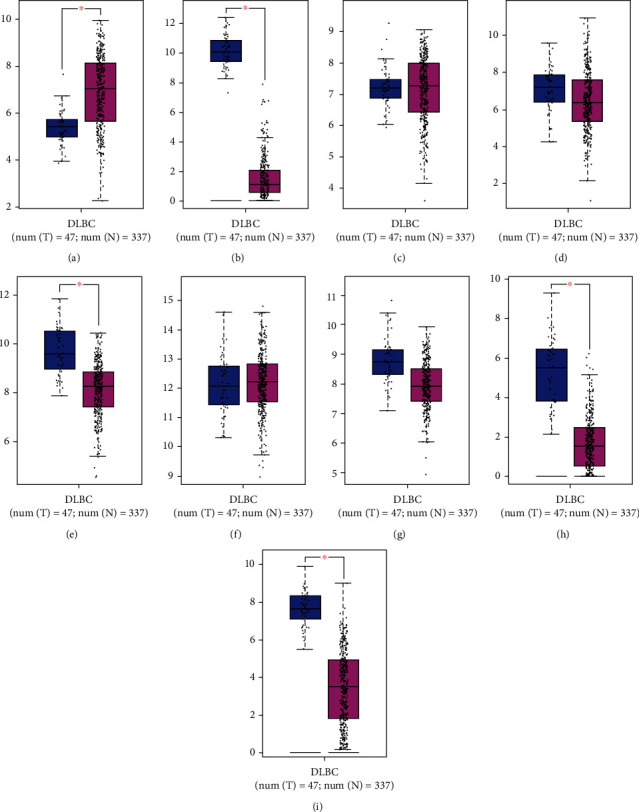
Expression of 9 hub genes in DLBC and normal controls: (a) *AGTRAP*, (b) *APOE*, (c) *BRI3*, (d) *CCL5*, (e) *CTSD*, (f) *FTH1*, (g) *GPX1*, (h) *LGALS2*, and (i) *TMEM176B*.

**Figure 9:**
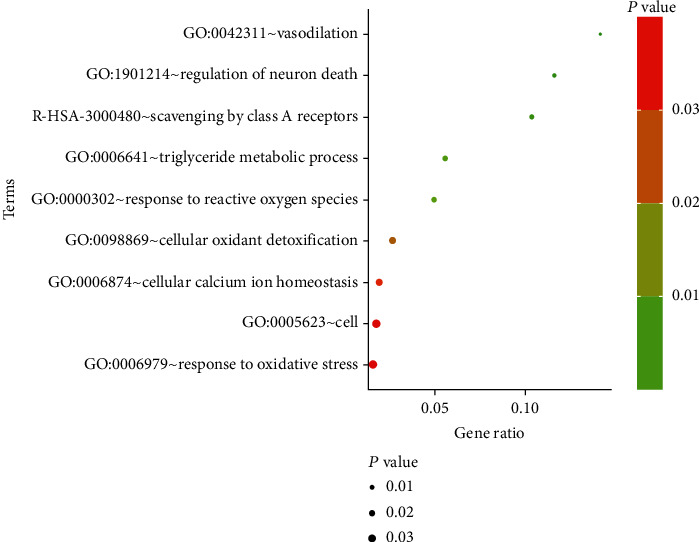
Results of gene ontology. ROS and oxidative stress are the main related function sets of enrichment.

**Figure 10:**
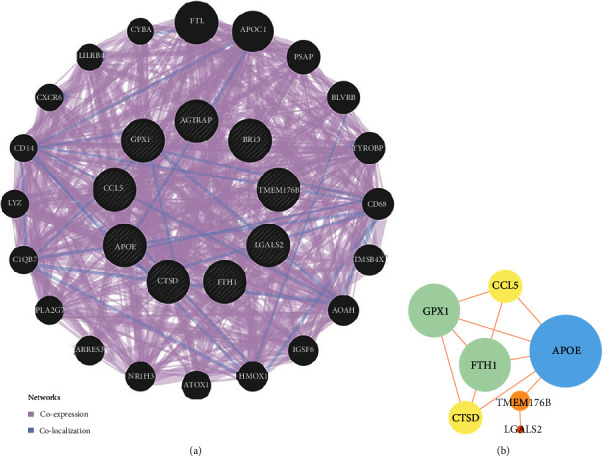
(a) GeneMANIA was used to build a genetic interaction network with a common goal. The black nodes with a slash represent the query gene, and the other nodes represent the prediction genes. (b) A physically and functionally connected protein-protein interaction network that implements common goals through strings. Nodes represent proteins, and edges represent pairs of interactions between proteins. Node size and color represent richness, while edge size and color represent combined scores.

The genes of the black module were introduced into Cytoscape software, and the top 10 hub genes (*GPX1*, *CTSD*, *TMEM176B*, *APOE*, *FTH1P20*, *AGTRAP*, *CCL5*, *BRI3*, *LGALS2*, and *FTH1*) were screened out by cytoHub tool (Figure 7). However, the *FTH1P20* is a pseudogene. It is a nonfunctional residue formed by the gene family in the process of evolution. It is similar to normal genes, but the loss of normal function of DNA sequences often exists in eukaryotes' polygene family [21]. Thus, *FTH1P20* was excluded from subsequent analysis. Then, we used GEPIA to express these nine hub genes in tumor tissues and nontumor tissues and found that the *CTSD*, *TMEM176B*, *APOE*, *AGTRAP*, and *LGALS2* expressions in tumor tissues and nontumor tissues were different (Figure 8). In addition, GO and KEGG analyses of these hub genes were mainly enriched at vasodilation (GO: 0042311), regulation of neuron death (GO: 1901214), triglyceride metabolic process (GO: 0006641), response to reactive oxygen species (GO: 0000302), cellular oxidant detoxification (GO: 0098869), cellular calcium ion homeostasis (GO: 0006874), response to oxidative stress (GO: 0006979), cell (GO: 0005623), and R-HSA-3000480 (Figure 9). GeneMANIA showed the GI network of hub genes interaction at the mRNA expression level. The STRING database generated the PPI coexpression network by analyzing the hub genes at the protein level (Figure 10).

### 3.3. Survival Analysis and Diagnostic Analysis

**Figure 11:**
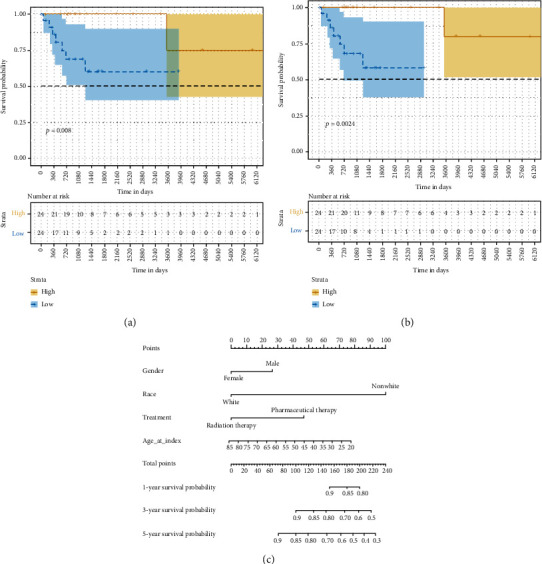
Prognostic significance of common index of overall survival in patients with diffuse large B-cell lymphoma. Kaplan-Meier survival curve showed that (a) *APOE* and (b) *CTSD* were significantly correlated with prognosis. A nomogram of the relationship between medical data and risk scores (c).

According to the log-rank test and Kaplan-Meier estimation, 2 of the 10 hub genes were significantly associated with OS: *APOE* (*P* = 0.008, Figure 11(a)) and *CTSD* (*P* = 0.0024, Figure 11(b)) in DLBC patients. According to the clinical data of the patients, a nomogram was generated (Figure 11(c)). The *c*-index of the model was 0.7.

### 3.4. mRNA Correlation Analyses

**Figure 12:**
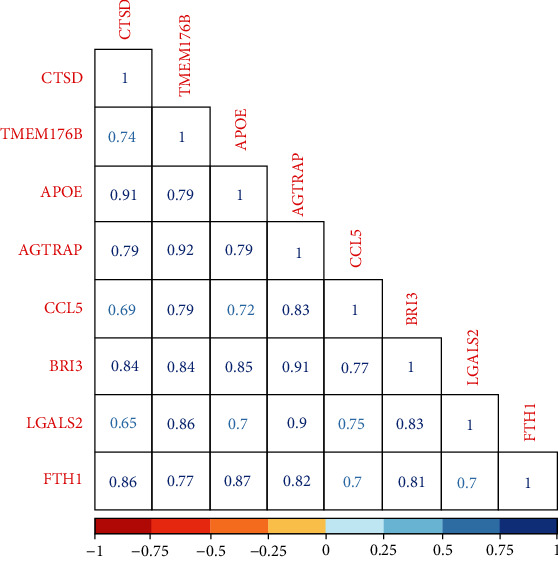
Relationship between mRNA expressions of hub genes.

The correlation of mRNA expression level of hub genes was determined by the Pearson correlation coefficient analysis. The results showed that all the 10 hub genes were significantly correlated (*P* < 0.01) with each other, and the correlation coefficients were all greater than 0.5 (Figure 12).

### 3.5. GSEA Results

**Figure 13:**
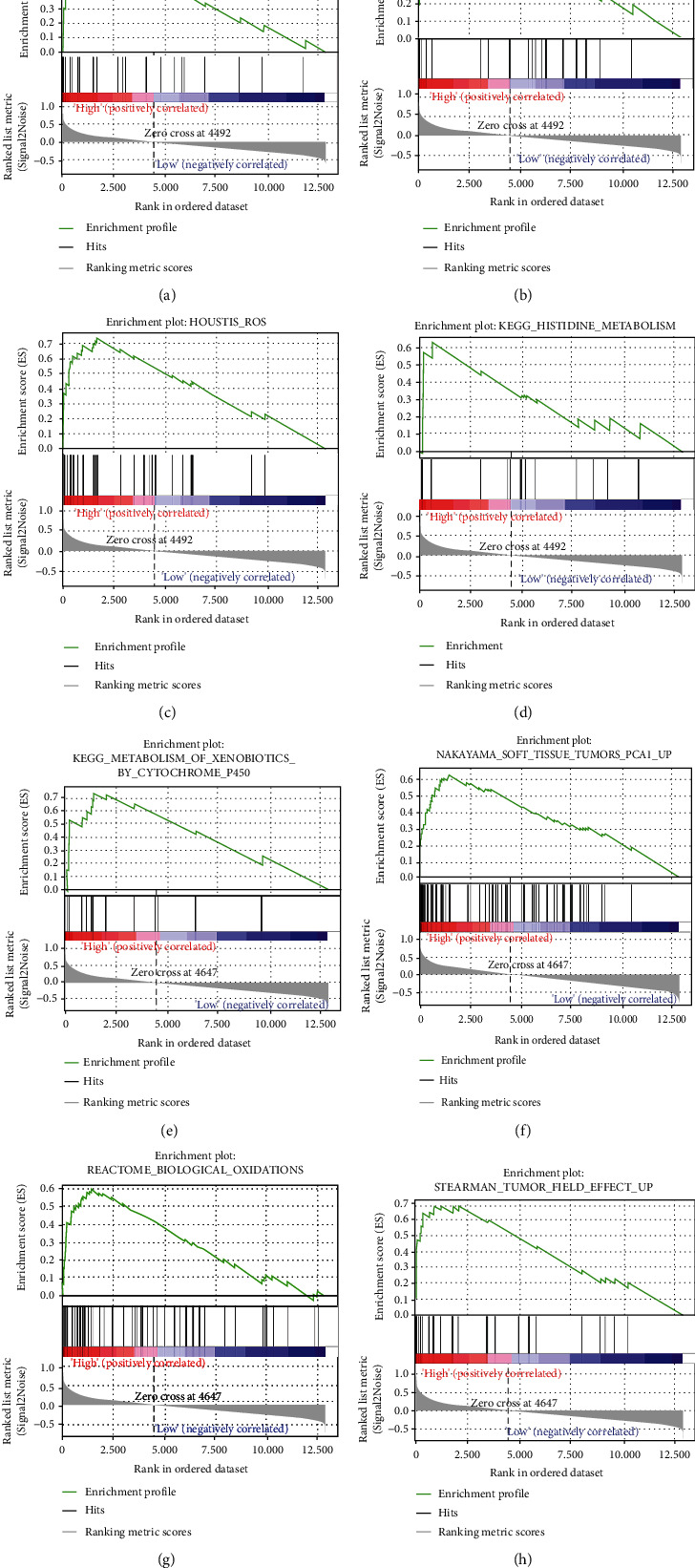
Gene set enrichment analysis enrichment plots for (a–d) *APOE* and (e–h) *CTSD*.

GSEA calculates the enrichment score (ES) by searching the gene list. When the gene is at the gene concentration, the score is increased, and when the gene is not at the gene concentration, the score is reduced. Positive and negative ES indicate that the gene set is enriched at the top or bottom of the ranking list, respectively. The gene before the peak is the core gene under the gene set. GSEA results showed that *APOE* was mainly enriched in fatty acid metabolism, and the regulation of ROS signaling pathways can regulate the body's metabolism and the microenvironment of tumor cells. In addition, *CTSD* could regulate tumor tissue, cytochrome metabolic pathway, and oxidative stress (Figure 13). In conclusion, the two prognosis-related genes *APOE* and *CTSD* were mainly related to regulating tumor and metabolism-related functions.

## 4. Discussion

DLBC is a heterogeneous malignant tumor in biology and clinic. Some biomarkers have been found in previous studies but have not been used in clinics [22]. In this study, 10 hub genes and several pathways were identified by WGCNA. GO and KEGG functional analyses of hub genes were carried out, and then, PPI and GI were used to verify the interaction at the protein and gene level. Among the hub genes, *APOE* and *CTSD* were proved as survival-affected genes. As a bioinformatics algorithm, WGCNA uses a coexpression network to establish genetic modules and screen possible pathogenesis or potential therapeutic targets [23]. So far, gene modules related to several cancers have been analyzed and verified by WGCNA [24, 25].

Fourteen modules were obtained in the training set in this study, one of which showed high stability. GO and KEGG analysis showed that mRNA in these four highly stable modules was mainly involved in regulating oxidative stress, ROS, and neuronal death, suggesting that they may be related to the pathogenesis of DLBC. Affecting prognosis has the potential to be used as a biomarker of cancer. It can assist in diagnosis, may become a therapeutic target in the future, and can also predict the survival probability of patients. It can even provide a theoretical basis for understanding the occurrence and development of a tumor and its molecular mechanism [26–28]. This study obtained the top 10 hub genes (*GPX1*, *CTSD*, *TMEM176B*, *APOE*, *FTH1P20*, *AGTRAP*, *CCL5*, *BRI3*, *LGALS2*, and *FTH1*) from the black module of WGCNA. PPI and GI analysis of these hub genes showed that they had related biological functions. Based on these hub genes, we analyzed their survival and Cox regression to analyze the effects of these genes on DLBC patients. It was found that *APOE* and *CTSD* affected the prognosis of patients. At the same time, it was found that age and race could affect the score of patients. Therefore, a new risk assessment system for DLBC patients can be established based on the above genes to help detect the high-risk groups of the disease.

Previous studies have found that both prognostic genes are associated with human cancer. Apolipoproteins E (*APOE*) gene has three main subtypes (E2, E3, and E4) polymorphism. These three subtypes can form six genetic combinations, and the structure and function of proteins formed by different combinations are different [29]. Because *APOE* regulates cholesterol levels, variation in lipoprotein E may explain its association with the risk of prostate cancer [30, 31]. In addition, the change of *APOE* level was earlier than that of the lymphedema index. Therefore, elevated blood APOE levels may be used to monitor the risk of lymphedema in breast cancer survivors [32]. Some studies have shown that Cathepsin D (*CTSD*) is a lysosomal aspartic protease, which can be used as a tumor marker. Because of its increased concentration in the cytoplasm of breast cancer cells, it is the most studied lysosomal aspartate protease [33, 34].

Furthermore, many immunohistochemical results showed that the enhanced expression of *CTSD* encoded protein was an indicator of the malignant degree of serous ovarian cancer [35]. Over the past two decades, research has shown that overexpression and hypersecretion of CTSD have increased in many types of cancer, including ovarian cancer and breast cancer, endometrial cancer, lung cancer, and cancer glioma, melanoma, and prostate cancer [36–41]. Hence, *CTSD* may be a promising biomarker for these tumors. Thus, the above two hub genes play a role in many tumors; they may also play a role in DLBC.

We further analyzed the genes in the hub genes that had an impact on survival analysis. It is found that *APOE* and *CTSD* play an essential role in many aspects of the tumor, such as cell adhesion, the effect of ROS, the regulation of oxidative stress, fat metabolism, and the regulation of tumor tissue. Studies have shown that increasing the expression of ROS can inhibit the growth of nasopharyngeal carcinoma cells [42, 43]. Moreover, the influence of cell energy and metabolism may affect the regulation of the tumor tissue microenvironment. Additionally, the latest studies showed that the change of oxygen concentration affects the cellular function of DLBC and overexpression of DNA damage markers caused by oxidative stress in cell subsets with a poor prognosis of DLBC [44, 45]. Therefore, these two hub genes may regulate the occurrence and development of tumors and affect patients' prognosis.

## 5. Conclusion

In summary, our study used TCGA database and identified and verified nine hub genes associated with the DLBC based on WGCNA. Survival analysis showed that the overexpression of *APOE* and *CTSD* in DLBC may be a poor prognostic indicator. At the same time, further studies *in vivo* and *in vitro* are needed to clarify its potential molecular mechanism. These findings provide a framework for identifying DLBC coexpression gene modules, identifying the key pathways and driving genes that may be new therapeutic targets, and contributing to the development of prognostic indicators.

## Data Availability

The data used to support the findings of this study are included within the article.
